# High-Sensitivity C-Reactive Protein and Risk of Sepsis

**DOI:** 10.1371/journal.pone.0069232

**Published:** 2013-07-23

**Authors:** Henry E. Wang, Nathan I. Shapiro, Monika M. Safford, Russell Griffin, Suzanne Judd, Joel B. Rodgers, David G. Warnock, Mary Cushman, George Howard

**Affiliations:** 1 Department of Emergency Medicine, University of Alabama School of Medicine, Birmingham, Alabama, United States of America; 2 Department of Emergency Medicine, Beth Israel Deaconess Medical Center, Boston, Massachusetts, United States of America; 3 Division of Preventive Medicine, Department of Medicine, University of Alabama School of Medicine Birmingham, Alabama, United States of America; 4 Department of Epidemiology, School of Public Health, University of Alabama at Birmingham, Birmingham, Alabama, United States of America; 5 Department of Biostatistics, School of Public Health, University of Alabama at Birmingham, Birmingham, Alabama, United States of America; 6 Division of Nephrology, Department of Medicine, University of Alabama School of Medicine, Birmingham, Alabama, United States of America; 7 Division of Hematology and Oncology, Department of Medicine, University of Vermont, Burlington, Vermont, United States of America; Virginia Tech University, United States of America

## Abstract

**Background:**

Conventional C-reactive protein assays have been used to detect or guide the treatment of acute sepsis. The objective of this study was to determine the association between elevated baseline high-sensitivity C-reactive protein (hsCRP) and the risk of future sepsis events.

**Methods:**

We studied data from 30,239 community dwelling, black and white individuals, age ≥45 years old enrolled in the REasons for Geographic and Racial Differences in Stroke (REGARDS) cohort. Baseline hsCRP and participant characteristics were determined at the start of the study. We identified sepsis events through review of hospital records. Elevated hsCRP was defined as values >3.0 mg/L. Using Cox regression, we determined the association between elevated hsCRP and first sepsis event, adjusting for sociodemographic factors (age, sex, race, region, education, income), health behaviors (tobacco and alcohol use), chronic medical conditions (coronary artery disease, diabetes, dyslipidemia, hypertension, chronic kidney disease, chronic lung disease) and statin use.

**Results:**

Over the mean observation time of 5.7 years (IQR 4.5–7.1), 974 individuals experienced a sepsis event, and 11,447 (37.9%) had elevated baseline hsCRP (>3.0 mg/L). Elevated baseline hsCRP was independently associated with subsequent sepsis (adjusted HR 1.56; 95% CI 1.36–1.79), adjusted for sociodemographics, health behaviors, chronic medical conditions and statin use.

**Conclusion:**

Elevated baseline hsCRP was associated with increased risk of future sepsis events. hsCRP may help to identify individuals at increased risk for sepsis.

## Introduction

Sepsis, the syndrome of microbial infection complicated by systemic inflammatory response, is a major public health problem associated with significant morbidity and mortality. [1] The national burden of severe sepsis care in the United States is substantial, encompassing an estimated 750,000 hospital admissions, 570,000 Emergency Department visits, 200,000 deaths and $16.7 billion in medical expenditures annually.[2]–[4] While efforts to reduce the public health impact of sepsis have focused primarily on optimizing hospital outcomes after the onset of disease, there has been relative little attention directed towards identifying its antecedent risk factors. [5], [6]

Biomarkers have been broadly used to aid in the diagnosis, prognostication or therapy of many medical conditions. [7] C-reactive protein (CRP) is an acute phase protein that plays a prominent role in many diseases. Elevated CRP levels detected using high-sensitivity assay techniques have been associated with risk of cardiovascular disease, stroke and all-cause mortality.[7]–[9] While often used to detect or guide the treatment of acute sepsis, there have been few efforts linking CRP level at a stable phase of health with risk of *future* sepsis events.[10]–[18] As with cardiovascular disease, if baseline CRP levels were associated with future sepsis events, this finding could motivate strategies to mitigate disease severity or mortality, or to prevent the onset of the condition.

The objective of this study was to determine the association between baseline high-sensitivity CRP (hsCRP) and future risk of sepsis in community-dwelling individuals.

## Materials and Methods

### Ethics Statement

This study was approved by the Institutional Review Board of the University of Alabama at Birmingham with waiver of the requirement for informed consent.

### Study Design

This study utilized data from the Reasons for Geographic and Racial Differences in Stroke (REGARDS) cohort, a national population-based longitudinal cohort.

### Selection of Participants

Designed to evaluate geographic and black-white stroke mortality variations, REGARDS is one of the largest ongoing national cohorts of community-dwelling individuals in the US, encompassing 30,239 individuals ≥45 years old. [19] REGARDS includes individuals from all regions of the continental US. Participant representation oversampled the Southeastern US, with 20% of the cohort originating from the coastal plains of North Carolina, South Carolina and Georgia (the “buckle” of the stroke belt), and 30% originating from the remainder of North Carolina, South Carolina and Georgia plus Tennessee, Mississippi, Alabama, Louisiana and Arkansas (the “stroke belt”). The cohort is 41% African American and 45% male, and 69% of individuals are >60 years old. The cohort does not include Hispanics where stroke mortality disparities are small to non-existent.

REGARDS enrolled participants during 2003–7. REGARDS obtained baseline data for each participant using both phone interview and in-person evaluations. Baseline data included medical history, functional status, health behaviors, physical characteristics (height, weight), physiologic measures (blood pressure, pulse, electrocardiogram), and an inventory of medications. Each participant provided blood and urine specimens. Self-administered questionnaires evaluated diet, family history of diseases, psychosocial factors and prior residences. On a semi-annual basis, the study contacted each participant to determine the date, location and attributed reason for all emergency department visits and hospitalizations during the follow-up interval. If the participant died, the study team reviewed death certificates and medical records and interviewed proxies to ascertain the circumstances of the participant’s death.

### Identification of Sepsis Events

Using a taxonomy for serious infections developed by Angus, et al., we reviewed all reported hospital admissions and Emergency Department visits attributed by participants to a serious infection. [2] Two trained abstractors independently reviewed all relevant medical records to confirm the presence of a serious infection on initial hospital presentation, and the relevance of the serious infection as a major reason for hospitalization. The abstractors identified clinical and laboratory information from the first 28-hours of hospitalization. The abstractors adjudicated discordances, with additional physician-level review as needed.

Consistent with international consensus definitions, sepsis consisted of presentation to the hospital with an infection plus two or more systemic inflammatory response syndrome (SIRS) criteria, including 1) heart rate >90 beats/minute, 2) fever (temperature >38.3°C or <36°C), 3) tachypnea (>20 breaths/min) or PCO_2_<32 mmHg, and 4) leukocytosis (white blood cells [WBC] >12,000 or <4,000 cells/mm^3^ or >10% band forms). [1] Presentation to the hospital consisted of the time of Emergency Department triage or admission to inpatient unit (for participants admitted directly to the hospital). To allow for acute changes in the participant’s condition during early hospitalization, we used vital signs and laboratory test results for the initial 28-hours of hospitalization. We did not include vital signs or laboratory findings at later time points of the hospitalization. We did not include sepsis developing at later time points of the hospitalization. We did not include organ dysfunction in the definition of sepsis. Initial review of 1,349 hospital records indicated excellent inter-rater agreement for presence of a serious infection (kappa = 0.92) and the presence of sepsis (kappa = 0.90) upon hospital presentation.

### Measurement of Baseline hsCRP

Trained personnel collected blood samples from all REGARDS participants at subjects’ homes following a 10–12 hour fast. Samples were centrifuged and serum or plasma separated within 2 hours of collection (mean 97 minutes, SD 127 minutes) and shipped overnight on ice packs to the central laboratory at the University of Vermont. Overnight shipping of samples was achieved for 95% of the cohort. On arrival, samples were centrifuged at 30,000 *g* and 4°C, and either analyzed (general chemistries) or stored at −80°C. hsCRP was determined in batches by particle-enhanced immunonephelometry using the BNII nephelometer (N High-sensitivity CRP; Dade Behring) with interassay CVs of 2.1%–5.7%. There was no difference in hsCRP distribution by number of sample shipping days. In contrast with conventional CRP assays, the high-sensitivity CRP technique is able to detect levels as low as 0.04 mg/L.

### Covariates

We considered covariates correlated with hsCRP including sociodemographic characteristics, health behaviors and chronic medical conditions. [20] Sociodemographic characteristics included age, sex, race, geographic region, self-reported annual household income and self-reported education (years of school). Geographic region was defined as participant residence in the stroke “buckle,” stroke “belt,” and elsewhere. [19] Health behaviors included tobacco and alcohol use. Smoking status use was defined as current, past and never. We defined alcohol use according to the National Institute on Alcohol Abuse and Alcoholism classification; i.e., moderate (1 drink per day for women or 2 drinks per day for men) and heavy alcohol use (>1 drink per day for women and >2 drinks per day for men). [21]

Evaluated chronic medical conditions included hypertension, diabetes, dyslipidemia, coronary artery disease, chronic kidney disease, and chronic lung disease. Hypertension consisted of systolic blood pressure ≥140 mm Hg, diastolic blood pressure ≥90 mm Hg, or the reported use of antihypertensive agents. Diabetes included a fasting glucose ≥126 mg/L (or a glucose ≥200 mg/L for those not fasting) or the use of insulin or oral hypoglycemic agents. Dyslipidemia consisted of low density lipoprotein cholesterol >130 mg/dL, or use of lipid lowering medications. A history of coronary artery disease consisted of individuals with a self-reported history of myocardial infarction, coronary intervention or baseline electrocardiographic evidence of myocardial infarction.

Chronic kidney disease consisted of an estimated glomerular filtration rate <60 ml/min/1.73 m^2^ calculated using the CKD-EPI equation. [22] Because REGARDS did not collect information on pulmonary conditions such as asthma and chronic obstructive pulmonary disease, we defined participant use of pulmonary medications as a surrogate for chronic lung disease. Obtained from each participant’s medication inventory, pulmonary medications included beta agonists, leukotriene inhibitors, inhaled corticosteroids, combination inhalers, and other pulmonary medications such as ipratropium, cromolyn, aminophylline and theophylline. We also identified statin use through the participant’s medication inventory.

### Data Analysis

To maintain consistency with cardiovascular studies, we defined elevated baseline hsCRP as >3.0 mg/L. [23] We compared demographic, health behavioral and clinical characteristics between individuals with normal and elevated hsCRP. Using Cox proportional hazards regression, we calculated hazard ratios (HR) and 95% confidence intervals (CI) for the association between elevated baseline hsCRP and first episode of sepsis during follow-up. We defined person-time at risk as the time (days) from first in-person examination to the first episode of sepsis or the last follow-up interview, whichever came first. We adjusted the models for demographic characteristics (age, sex, race, education, geographic region, income), health behaviors (smoking and alcohol use), and chronic medical conditions (hypertension, dyslipidemia, coronary artery disease, chronic kidney disease, chronic lung disease). Because statins lower inflammatory markers, we also adjusted for statin use. [24]

Due to the time lag in observations and medical record retrieval, we could not review medical records for 1,157 participants with reported hospitalizations for a serious infection over the observation period (2003–2011), a figure expected to yield an additional 300 sepsis events based on adjudication rates for other participants. Therefore, we repeated the analysis excluding these individuals. The primary analysis focused on sepsis events. In a secondary analysis we examined the association between hsCRP and non-sepsis infection events (serious infections that did not fulfill SIRS criteria). We conducted all analyses using SAS v9.3.

## Results

Among the 30,239 REGARDS participants, from February 5, 2003 through July 30, 2012, we identified 2,151 hospitalizations for a serious infection, encompassing 1,179 sepsis events among 974 individuals. Mean follow-up time was 5.7 years (IQR 4.5–7.1).

Among participants with available values, median hsCRP was 2.2 mg/L (IQR 1.0, 5.0), and 11,447 (37.9%) exhibited elevated hsCRP (>3.0 mg/L). Among the 974 sepsis individuals with hsCRP levels available, the most common infection types associated with the first sepsis episode were pneumonia, kidney and urinary tract infections, and abdominal infections. (Table 1) Individuals with elevated hsCRP were more likely to be younger, female, or black, and to report lower income and education. (Table 2) While individuals with elevated hsCRP were more likely to be current or past smokers, they were less likely to report heavy or moderate alcohol use. Chronic medical conditions were more common in individuals with elevated hsCRP.

**Table 1:** Infection types associated with hospitalizations for sepsis.

Infection Type	Number of Sepsis Hospitalizations (n = 974) N (%)
Pneumonia	416 (42.7)
Kidney and Urinary Tract Infections	157 (16.1)
Abdominal	136 (14.0)
Bronchitis, Influenza and other Lung Infections	89 (9.1)
Skin and Soft Tissue	74 (7.6)
Sepsis	58 (6.0)
Fever of Unknown Origin	15 (1.5)
Surgical Wound	7 (0.7)
Catheter (IV/Central/Dialysis)	5 (0.5)
Meningitis	3 (0.3)
Unknown/Other	14 (1.4)

Includes first sepsis episodes only.

**Table 2:** Baseline characteristics of subjects with normal ≤3.0 mg/L and elevated >3.0 mg/L baseline high-sensitivity C-reactive protein (hsCRP).

Characteristic	hsCRP≤3.0 mg/L (n = 18,736)	hsCRP>3.0 mg/L (n = 11,447)	p-value*
**DEMOGRAPHICS**			
**Age (%)**			
45–61	38.2	40.7	<0.001
62–67	22.3	23.6	
68–75	24.0	22.3	
76+	15.5	13.4	
**Gender (%)**			
Male	50.2	36.2	<0.001
Female	49.8	63.8	
**Race (%)**			
White	63.2	50.9	<0.001
Black	36.8	49.1	
**Education (%)**			
Less than high school	10.8	15.6	<0.001
High school graduate	24.8	27.7	
Some college	26.2	27.9	
College or higher	38.3	28.9	
**Income (%)**			
<$20 k	15.5	22.5	<0.001
$20 k–$34 k	23.1	26.0	
$35 k–$74 k	30.6	27.8	
≥$75 k	18.3	11.6	
Refused/unknown	12.5	12.1	
**Geographic Region (%)**			
Stroke Buckle	20.3	21.9	<0.001
Stroke Belt	34.2	35.4	
Non-Belt/Buckle	45.5	42.8	
**HEALTH BEHAVIORS**			
**Tobacco Use (%)**			
Current	12.2	18.5	<0.01
Past	40.7	39.2	
Never	47.1	42.3	
**Alcohol Use (%)**			
Heavy	4.3	3.6	<0.001
Moderate	35.9	29.0	
None	59.8	67.4	
**CHRONIC MEDICAL CONDITIONS**			
Chronic Kidney Disease (%)	23.2	27.1	<0.001
Chronic Lung Disease (%)	8.0	11.2	<0.001
Coronary Artery Disease (%)	17.6	18.6	0.03
Diabetes (%)	19.6	25.8	<0.001
Dyslipidemia (%)	34.6	31.8	<0.001
Hypertension (%)	54.8	66.7	<0.001
Statin use (%)	32.9	28.9	<0.001

* Based on chi-square test.

Among REGARDS participants, 491 of 18,736 (2.6%) individuals with hsCRP≤3.0 mg/L developed sepsis, and 483 of 11,447 (4.2%) with CRP>3.0 mg/L developed sepsis. Elevated baseline hsCRP (>3.0 mg/L) was independently associated with an increased risk of sepsis (HR 1.68; 95% CI: 1.48–1.90). (Table 3) Elevated baseline hsCRP remained independently associated with sepsis risk after adjustment for demographic characteristics, health behaviors, chronic medical conditions and statin use (HR 1.56; 95% CI 1.36–1.79). (Table 3).

**Table 3:** Hazard ratios (HRs) and 95% confidence intervals (CI) for the adjusted associations between elevated baseline high-sensitivity C-reactive protein (hsCRP>3.0 mg/L) and first sepsis episodes.

Model	N	Events	Hazard Ratio (95% CI)
Crude (unadjusted) HR	29,667	972	1.68 (1.48–1.90)
Adjusted for age, sex, race, region	29,667	972	1.86 (1.64–2.12)
Additional adjustment for education and income	29,644	970	1.77 (1.56–2.01)
Additional adjustment for smoking and alcohol use	28,959	940	1.70 (1.50–1.94)
Additional adjustment for chronic medical conditions* and statin use	26,999	881	1.56 (1.36–1.79)

Total of 30,183 REGARDS participants included in the analysis.

* Coronary artery disease, diabetes, dyslipidemia, hypertension, chronic kidney disease, chronic lung disease.

When repeating the analysis excluding the 1,157 participants with unadjudicated potential sepsis events, there was a similar association between CRP and incident sepsis (adjusted HR 1.51; 95% CI 1.31–1.76). Excluded individuals were less likely to be African American (39.6 vs. 41.5%) and exhibited slightly higher hsCRP levels (mean 5.4 vs. 4.6 mg/L). In a secondary analysis we identified 828 individuals with non-sepsis infection events (serious infections that did not fulfill SIRS criteria). While elevated baseline hsCRP was independently associated with the risk of non-sepsis infection events (adjusted HR 1.37; 95% CI: 1.18–1.60), the relationship was lower than that for sepsis events.

## Discussion

Elevated hsCRP levels detected using high-sensitivity assay techniques have been associated with risk of cardiovascular disease, stroke and all-cause mortality.[7]–[9] In this study of individuals in the REGARDS cohort, we found that elevated baseline hsCRP was associated with increased risk of future sepsis events. This finding suggests that knowledge of hsCRP level could help to identify individuals at increased risk for sepsis.

There are plausible pathophysiologic connections between elevated baseline hsCRP and future sepsis events. CRP is believed to activate complement, interact with cell surface receptors, induce a prothrombotic state, increase expression of inflammatory mediators, and upregulate endothelial cell adhesion molecules, among other actions. [25] Many of the same inflammatory processes also play key roles in sepsis. [2], [26] For example, we previously identified associations between inflammatory and endothelial activation biomarkers and future sepsis events. [27] Collectively, these observations suggest that elevated hsCRP may signal individuals with a chronic low-grade proinflammatory state and prone to the hyperinflammatory state characteristic of sepsis. An important question requiring additional study is whether hsCRP exerts a direct effect upon sepsis susceptibility or represents a marker of the inflammatory process that is causal in increasing sepsis risk.

Prior studies have associated acute CRP increases (on the order of 10–200 mg/dL) as a marker of sepsis illness severity as well as predicted of sepsis outcomes.[10]–[18] For example, in a series of 50 critically ill sepsis patients, Schmit, et al. observed that CRP on hospital admission was 16.7±10.6 mg/dL and that the magnitude of CPR decrease was associated with response to antimicrobial therapy. [18] Povoa, et al. studied 891 intensive care unit patients with community-acquired sepsis, observing a mean hospital admission CRP level of 20.1±13.9 mg/dL and finding association between rates of CRP decline and hospital survival. [13] In contrast, our findings are based upon levels determined at a stable phase of health using a *high-sensitivity* CRP assay, which detects levels as low as 0.04 mg/L. In addition, we focused upon the association between *baseline* hsCRP levels and risk of *future* sepsis events – not the acute course of illness.

Prior studies have examined the association between CRP levels and infection risk. For example, in a case-control study of 144 patients with HIV infection, Bjerk, et al. found that higher hsCRP was associated with a two-fold increased odds ratio for pneumonia. [17] In a study of 3,075 elderly (>70 years old) participants in the Health ABC cohort, Yende, et al. found that higher baseline CRP levels were not independently associated with pneumonia risk. [28] However, Yende’s effort did not utilize the newer high sensitivity CRP assay, as we used in the current study. We note that in the current study we did identify an independent association between baseline hsCRP and non-sepsis infection risk. While we did not separately examine pneumonia cases, over half of the sepsis cases in this series were due to lung infections.

We emphasize that we did not set out to develop a sepsis risk prediction rule using hsCRP. Our study merely indicates the presence of an association between hsCRP and future sepsis risk. However, given the results of this study, hsCRP may potentially play a role in a formal derivation of a risk prediction model. If the risk predictive abilities of hsCRP were validated, there could be plausible strategies to reduce the risk or impact of sepsis. For example, individuals at high risk of sepsis may merit more proactive treatment at the earliest stages of microbial infection, with earlier initiation of antibiotics, reduced thresholds for hospital admission, more aggressive early resuscitation, or more vigilant laboratory and physiologic monitoring during hospitalization. Statin therapy reduces cardiovascular events in individuals with elevated baseline hsCRP. [29] Gupta, et al. showed that statin use was associated with a reduced risk of hospitalization for sepsis among individuals with chronic kidney disease. [30] Other studies of hospitalized sepsis patients have suggested mitigated disease severity and organ dysfunction among those with pre-existing statin use. [31]

Limitations of this study should be noted. To maintain consistency with prior cardiovascular studies, in this study we defined elevated baseline hsCRP as >3.0 mg/L. [20] However, other hsCRP cutpoints are potentially possible for different age, sex or racial groups and merit additional study. Due to time lags in event reports and record retrieval, we have not yet reviewed medical records for a portion of individuals with reported serious infection hospitalizations. Furthermore, compared with individuals included in the analysis, excluded individuals were more likely to be black and to exhibit higher hsCRP levels. Therefore, our reported HRs may represent underestimates. Because REGARDS is not a surveillance study, we may not have detected all sepsis events. We did not examine severity variants of sepsis such as severe sepsis and septic shock because these conditions often develop later in the hospital course; however, it is possible that associations between hsCRP and various forms of sepsis may differ than those reported here. We also did not examine associations between hsCRP and repeat sepsis events.

By design, the REGARDS cohort includes only African Americans and whites, and thus we could not examine associations among other racial groups or Hispanic ethnicity. History of cancer was not ascertained by REGARDS, and this may represent an important risk factor for subsequent sepsis. Also, our study was able to detect the presence of chronic medical conditions but not their level of severity. Because the study did not collect information on pulmonary conditions, we used pulmonary medications as a surrogate for chronic lung disease. Our analysis utilized baseline hsCRP measurements; we could not assess the effect of changes of hsCRP over time. While we observed an association between elevated hsCRP and sepsis risk, elevated hsCRP may reflect risk for a host of inflammatory conditions not limited to sepsis; for example, cardiovascular disease and stroke. [8], [9]

### Conclusion

In this study elevated hsCRP at a stable phase of health was associated with increased risk of future sepsis events. hsCRP may identify individuals at increased risk for sepsis.
